# Identifying Tightly Regulated and Variably Expressed Networks by Differential Rank Conservation (DIRAC)

**DOI:** 10.1371/journal.pcbi.1000792

**Published:** 2010-05-27

**Authors:** James A. Eddy, Leroy Hood, Nathan D. Price, Donald Geman

**Affiliations:** 1 Institute for Genomic Biology, University of Illinois, Urbana, Illinois, United States of America; 2 Department of Bioengineering, University of Illinois, Urbana, Illinois, United States of America; 3 Institute for Systems Biology, Seattle, Washington, United States of America; 4 Center for Biophysics and Computational Biology, University of Illinois, Urbana, Illinois, United States of America; 5 Department of Chemical and Biomolecular Engineering, University of Illinois, Urbana, Illinois, United States of America; 6 Institute for Computational Medicine, Johns Hopkins University, Baltimore, Maryland, United States of America; 7 Department of Applied Mathematics and Statistics, Johns Hopkins University, Baltimore, Maryland, United States of America; KAIST, Republic of Korea

## Abstract

A powerful way to separate signal from noise in biology is to convert the molecular data from individual genes or proteins into an analysis of comparative biological network behaviors. One of the limitations of previous network analyses is that they do not take into account the combinatorial nature of gene interactions within the network. We report here a new technique, Differential Rank Conservation (DIRAC), which permits one to assess these combinatorial interactions to quantify various biological pathways or networks in a comparative sense, and to determine how they change in different individuals experiencing the same disease process. This approach is based on the *relative* expression values of participating genes—i.e., the ordering of expression within network profiles. DIRAC provides quantitative measures of how network rankings differ either among networks for a selected phenotype or among phenotypes for a selected network. We examined disease phenotypes including cancer subtypes and neurological disorders and identified networks that are tightly regulated, as defined by high conservation of transcript ordering. Interestingly, we observed a strong trend to looser network regulation in more malignant phenotypes and later stages of disease. At a sample level, DIRAC can detect a change in ranking between phenotypes for any selected network. Variably expressed networks represent statistically robust differences between disease states and serve as signatures for accurate molecular classification, validating the information about expression patterns captured by DIRAC. Importantly, DIRAC can be applied not only to transcriptomic data, but to any ordinal data type.

## Author Summary

The systems approach to medicine derives from the idea that diseased cells arise from one or more perturbed biological networks due to the net effect of interactions among multiple molecular agents; by measuring differences in the abundance of biomolecules (e.g., mRNA, proteins, metabolites) we can identify reporters of network states and uncover molecular signatures of disease. However, a major limitation of previously published network analyses is the focus on small numbers of individual, differentially-expressed genes, hence the failure to take into account combinatorial interactions. We report a new technique, Differential Rank Conservation, for identifying and measuring *network-level* perturbations. Our rank conservation index is based entirely on the *relative levels of expression* for participating genes and allows us to detect differences in network orderings between networks for a given phenotype and between phenotypes for a given network. In examining cancer subtypes and neurological disorders, we identified networks that are tightly and loosely regulated, as defined by the level of conservation of transcript ordering, and observed a strong trend to looser network regulation in more malignant phenotypes and later stages of disease. We also demonstrate that variably expressed networks represent robust differences between disease states.

## Introduction

Molecular signatures based on the measured abundance of biomolecules (e.g., mRNA, proteins, metabolites) have the potential to discriminate among disease subtypes, to predict clinical outcomes, or to provide insights into the mechanistic underpinnings of disease progression. Moreover, with sufficient data, these signatures begin to enable the identification of perturbed networks that reflect core aspects of the disease process—and thus could provide insights into functionally relevant drug targets as well as new approaches to diagnostics [1], [2]. However, distinguishing signal from noise in high-throughput data such as mRNA microarray experiments presents a significant challenge. This noise commonly results from technical issues in data production and the integration of datasets from different platforms, laboratories, or even experiments within a lab. Noise in high-throughput data also stems from biological variability in the sources, such as genetic polymorphisms, different stages of the biological process, disease stratification, and stages of disease progression. In the study of human disease processes, this variability poses a unique hurdle as there are often only data for a single point in time; when comparing data between individuals who appear to have the same disease, one does not know whether the observed differences reflect disease subtypes or different stages of a single disease type.

A fundamental tenant of systems approaches to biology and medicine is that dynamically changing biological networks mediate physiological, developmental, and disease processes, and that the key to understanding these processes is translating network dynamics into phenotypes. As such, a powerful method to mitigate some forms of biological noise (hence increasing the utility of high-throughput data as a diagnostic and scientific tool) is to convert the molecular data from individual genes or proteins into an analysis of comparative biological network behaviors. Typically, studies search for a small number of individual genes whose differential expression is highly correlated with phenotypic changes. However, malignant phenotypes in many diseases arise from the net effect of interactions among multiple genes and other molecular agents within biological networks. For example, cooperating oncogenes interact synergistically to evade tumor suppression mechanisms such as cell-cycle arrest and apoptosis [3], [4]. The combinatorial nature of such disease-induced perturbations leads to a highly complex picture of the underlying biological processes. As such, the biological insight gleaned from the expression patterns of individual genes is often limited. Other pitfalls associated with individual gene expression analysis have been proposed and discussed elsewhere [2], [5], [6].

The importance of studying network behavior—evident in most phenotypes, disease or otherwise—is particularly well-documented for cancer. Research has linked modulated function on the level of either metabolic networks [7]–[9] and/or signaling networks [10]–[12] to cancer hallmarks including angiogenesis, increased growth, metastasis, and evasion of immune detection. Similarly, recent global genomic analyses in glioblastoma multiforme [13], [14] and pancreatic cancers [15] have revealed both varying numbers and frequencies of genetic alterations within distinct core networks of each disease. In light of these findings, microarray data analysis methods have begun to shift towards identifying biologically meaningful pathways or networks. We consider all pathways to in fact be part of interconnected biological networks, and henceforth use the term network rather than pathway. In general, network regulation controls the expression levels of related genes responding to specific conditions. Existing tools for network-based expression analysis commonly investigate informative patterns of up-regulation or down-regulation (i.e., increases or decreases in expression) of genes in different disease states. For example, the widely-used gene set enrichment analysis (GSEA) platform identifies networks that are significantly enriched for individual genes that are highly correlated with a phenotype [5], [16]. Other methods employ a single statistic to represent the collective activity of a network (e.g., mean or median gene expression) [2], [17]; perturbed levels of network activity (i.e., collective up- or down-regulation) are then examined to identify those networks most differentially expressed between phenotypes. These frameworks have been applied to diverse cancer systems and serve as a robust source of biological discovery [2], [18].

Studying cellular regulation of networks in terms of “unidirectional” changes may, however, overlook subtle, yet influential, changes in the *relationships* among the genes within a network. This drawback directly reflects the combinatorial operation of genes in networks, in which the actions of one gene greatly influences the actions of other genes. By accounting for these combinatorial interactions we can begin to alleviate the signal-to-noise issues in disease-perturbed networks (as well as dynamically changing networks mediating physiology or development). In particular, even the elementary interactions captured by the relative orderings among two or three genes have been shown to provide powerful biomarkers for separating phenotypes [19]–[21]. With methods that aim to identify statistically significant up- or down-regulation of genes or networks, results will also depend largely on the context of the microarray experiment. Cellular regulation in a case with a number of up- or down-regulated genes in one phenotype versus another manifests as an increase in absolute expression levels above some threshold, relative to all other genes on the microarray. Even when thresholds are tuned to produce statistically significant results, the findings are still based on indirect measurements, (i.e., fluorescence) and therefore may depend heavily on the experimental set up, type of data normalization, and other factors. In addition to the technical limitations of microarray experiments, biological context can greatly impact results. For instance, if nearly all genes are differentially expressed between two phenotypes, then no single network will be statistically “enriched” for change. It is also possible that neither individual network genes nor any network as a whole will display notable over- or under-expression in response to environmental or disease-related stimuli. The importance of accounting for combinatorial gene interactions—and to do so without need to reference all of the genes on the microarray—again becomes clear.

We have developed a new method called Differential Rank Conservation (DIRAC) which considers combinatorial behavior, and provides quantitative measures of how network expression differs within and between phenotypes. The DIRAC approach assesses cellular regulation of a network in the context of the *relative levels of expression* for participating genes. For each microarray, the expression values of the network genes are ordered from highest expression (ranked first) to lowest expression (ranked last); regulation is then quantified entirely by the *rankings* of genes within a selected network. Consequently, DIRAC identifies and measures network-level perturbations from a completely novel perspective, namely the “combinatorial comparisons” of network genes as opposed to increases or decreases alone, allowing one to study how this ordering changes in different conditions—and thus begin to infer the consequences of combinatorial gene interactions. As a result, this approach has two key advantages over tools that measure absolute changes in expression levels. First, it accounts for gene-gene interactions; second, the results do not depend on the other genes on the microarray or on the method of normalization used. These are both critical points in dealing with signal-to-noise issues. Notably, as DIRAC treats each network independently, it can still identify perturbed networks even when every gene on the microarray is differentially expressed (in contrast to enrichment measures).

Our strategy for representing network rankings uses pairwise comparisons of gene expression levels. Such pairwise comparisons can yield two-gene predictors with simple decision rules for classification of expression profiles [22], [23]. These decision rules have in turn resulted in highly accurate two-gene diagnostic classifiers based on relative expression reversals that have proven effective for molecular identification of cancer [19]–[23]. We extend the relative expression reversal concept to networks. However, analyzing sample-to-sample changes for every possible distinct ordering of gene expression values within a network is not computationally feasible; there are simply too many possible orderings, i.e., permutations. Knowing the states of all pairwise orderings is equivalent to knowing the full ranking, which motivates our representation. For each distinct pair of genes within a network, we consider a binary variable indicating whether or not the mRNA abundance of the first gene is less than that of the second gene; in fact, we restrict attention to the probability of this event within a phenotype for each pair of genes. In this way, we avoid the combinatorial complexity of permutations and represent the “expected” ordering of network genes for a given phenotype as a binary template. Unlike the probabilities of full orderings, pairwise frequencies are reliably estimated with typical sample sizes, while still capturing a great deal of information about network regulation. We subsequently compute a *matching score* to signify how closely each sample's network ordering matches a phenotype-specific template.

We can use DIRAC at the population level to quantify conservation differences between networks for a given phenotype. Specifically, DIRAC allows us to use rankings to identify and contrast tightly and loosely regulated network types of a single phenotype:

- a network is considered *tightly regulated* within its phenotype if the ranks of network genes are mostly unchanged among samples;
- a network is considered *loosely regulated* if the ranks of network genes are greatly varied between samples of the same phenotype.

Tightness of regulation for a selected network is best understood as the allowed variation in gene expression levels observed across the population. This offers an advantage over studying up- or down-regulation only because it indicates the level of control across samples in a population. In this work we use the DIRAC approach to identify networks that are tightly regulated in a number of human cancers and neurological disorders. Since networks under tight control in a particular phenotype may be necessary to maintain a specific cellular function, tightly regulated networks that change across phenotypes may provide insight into processes such as disease progression.

Additionally, DIRAC can be applied at the sample level to identify conservation differences between phenotypes for a specified network. At this level the DIRAC method can identify variably expressed networks that reveal statistically robust differences between disease states, leading to highly accurate classification of expression profiles from various diseases. When used to separate expression profiles, the DIRAC method is noteworthy because it (i) is independent of microarray data normalization; (ii) results in a simple yet efficient classifier for phenotype distinction; and (iii) appears to be comparable in accuracy to state-of-the-art classification methods. Learning the regulation of gene rankings within different states allows us to discover molecular signatures composed of related genes that distinguish phenotypes, identify networks most involved in disease transitions, and assist identification of potential therapeutic targets. Importantly, while we focus on gene expression in the present study, the method can be generalized to any ordinal dataset, and thus can be applied to such biological data types as proteomics, gene copy number, chromosomal position, and so forth.

## Results/Discussion

### Overview of DIRAC Methods

**Figure 1:**
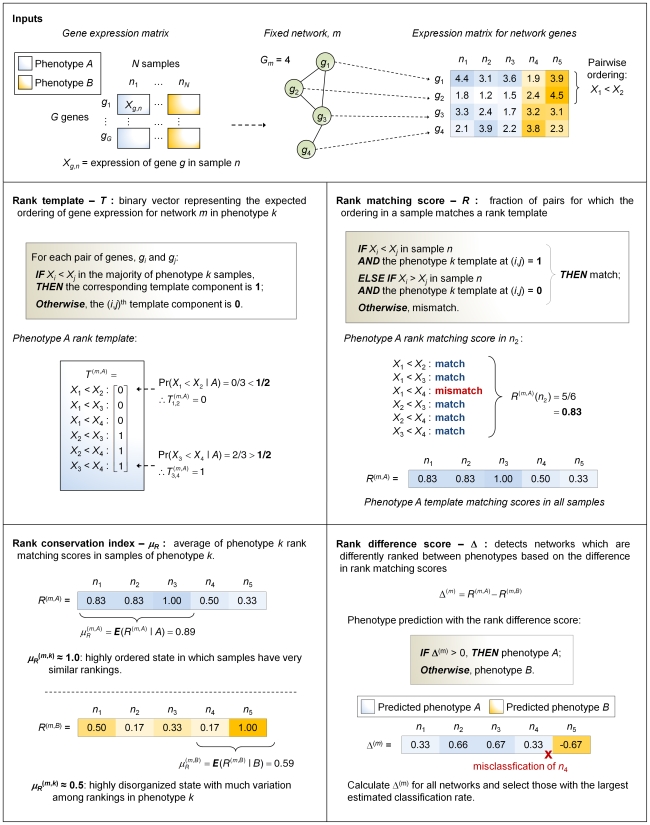
Overview of Differential Rank Conservation (DIRAC) methods.

The DIRAC approach was used to evaluate regulation of gene ordering within networks in different diseases. For each microarray sample in each phenotype studied, we characterized the ordering of network genes (i.e., network ranking) in terms of comparisons between the expression values of pairs of genes. Based on the comparison statistics, we defined a *rank template* for each network and phenotype representing the expected (i.e., most common) pairwise ordering of gene expression for that network in that phenotype. We employed a simple measure—a *rank matching score* (*R*)—to determine how well the network ranking in each individual sample (i.e., expression profile) matched the ordering defined in the rank template. Averaging *R* over all samples within a phenotype yields a network-specific *rank conservation index* (*μ_R_*) which represents how well, *on average*, all samples in the same phenotype match the corresponding rank template. Alternatively, comparing two rank matching scores for the same sample leads to a highly-discriminating *rank difference score* (Δ) that allows one to determine the most variably expressed networks between two phenotypes. The calculation of these quantities is illustrated in **Figure 1**.

**Figure 2:**
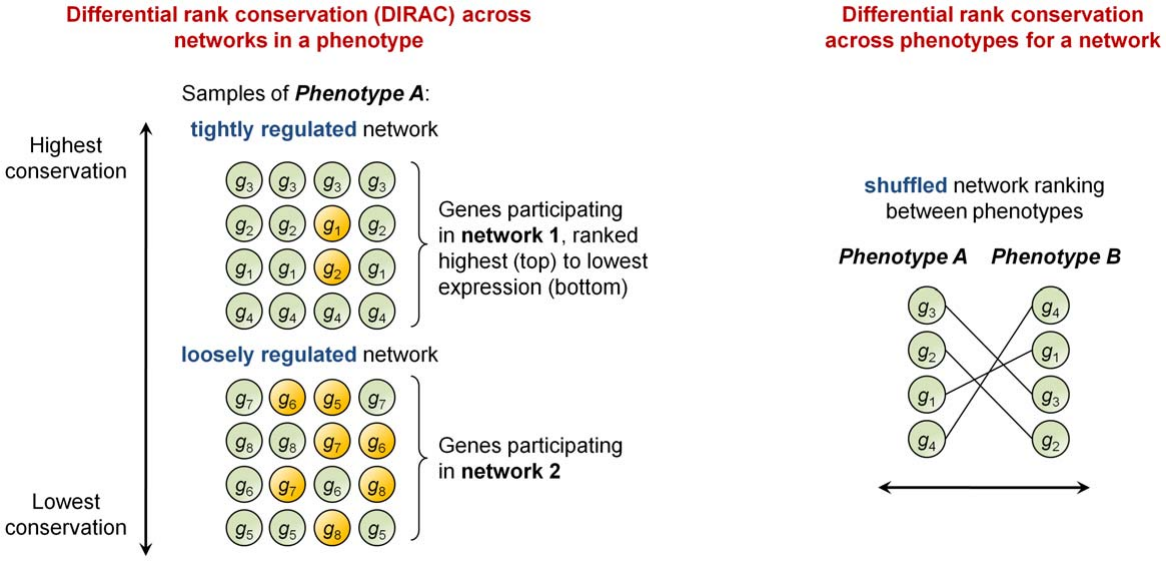
Prototypical scenarios observed for networks in DIRAC.

Several prototypical scenarios arise from these measures. In one scenario (**Figure 2** **, left**), conservation indices are used to measure the consistency with which network rankings are maintained in a population, and are used to identify tightly regulated networks in each phenotype. One situation, where all samples have similar network rankings, yields a large rank conservation index and indicates the network is tightly regulated. A second situation, where the ordering of network genes is highly varied, yields a small rank conservation index and indicates the network is loosely regulated. In a second prototypical scenario, the DIRAC method detects changes in ranking (i.e., shuffling of gene expression values) between phenotypes for a selected network (**Figure 2** **, right**). The top networks selected by DIRAC based on the difference score can be used to classify gene expression profiles by phenotype.

We first applied DIRAC to investigate network rankings using gene expression profiles obtained from patients with different stages of prostate disease. The gene expression data, originally reported by Yu et al. [24] and publically available in the NCBI Gene Expression Omnibus (GDS2545), contains 108 human prostate samples: 18 samples of normal prostate tissue (NP) from organ donors, 65 primary prostate tumor (PT) samples, and 25 metastatic prostate tumor (MT) samples. The findings for normal prostate and prostate cancer samples presented below represent the main features of the DIRAC method, and can be similarly obtained for any disease expression data.

In addition to the more detailed prostate cancer analysis, we examined a number of other disease phenotypes including cancer subtypes and neurological disorders, and identified both tightly regulated and variably expressed networks in each. For each dataset, we grouped expression levels of genes into 248 human signaling networks, defined according to the BioCarta gene sets collection in the Molecular Signatures Database (MSigDB) [5]. In order to ensure that the networks examined were as complete as possible, we used gene synonym information from NCBI to replace unmatched names in each dataset with those belonging to networks in the BioCarta collection. This step led to an average increase of 5% in the fraction of network genes (1296 total across 248 networks) for which a corresponding expression value was found (**Table S1**).

### Population-Level DIRAC

The population-level analysis is centered on the rank conservation index (*μ_R_*)-defined for each network and each phenotype. This index represents the degree of conservation in the rankings of the expression levels of the network genes, averaged over samples of the phenotype.

#### Tightly regulated networks in normal prostate and cancerous prostate

For a given phenotype, the extent of gene ranking conservation within networks will vary across networks. The ten most tightly regulated networks in normal prostate (NP), primary prostate tumors (PT), and metastatic prostate tumors (MT), as measured by rank conservation indices, are shown in **Table 1**. Large rank conservation index values indicate similar gene orderings among all samples of each phenotype in these networks, and hence tight regulation. This suggests that the combinatorial gene interactions in each network are quite similar among different patients.

**Table 1:** Most tightly regulated networks in normal prostate and primary and metastatic prostate tumors, as indicated by rank conservation index values.

Tightly regulated networks in NP				
Network name	Num. genes	Num. gene pairs^a^	Avg. variance in NP	*μ_R_* in NP
GS	6	15	1.328	1.000
FOSB	4	6	1.141	0.981
AKAP13	7	21	0.796	0.955
AGPCR	11	55	0.811	0.955
RNA	8	28	0.453	0.948
CACAM	12	66	0.551	0.947
NDKDYNAMIN	17	136	0.619	0.946
ETC	8	28	0.350	0.946
SET	11	55	0.537	0.945
SKP2E2F	10	45	0.339	0.943

Tightly regulated networks in PT				
Network name	Num. genes	Num. gene pairs	Avg. variance in PT	*μ_R_* in PT
GS	6	15	1.270	0.989
FOSB	4	6	1.525	0.979
AKAP13	7	21	0.880	0.960
ARGININEC	6	15	0.548	0.960
PLK3	8	28	0.672	0.951
CDC42RAC	15	105	0.547	0.946
RNA	8	28	0.489	0.946
CREM	7	21	0.563	0.944
BOTULIN	4	6	0.850	0.944
AGPCR	11	55	0.771	0.943

Tightly regulated networks in MT				
Network name	Num. genes	Num. gene pairs	Avg. variance in MT	*μ_R_* in MT
GS	6	15	1.322	0.995
FOSB	4	6	1.575	0.980
CREM	7	21	0.659	0.966
S1P	6	15	0.465	0.963
RAN	5	10	0.371	0.960
SLRP	4	6	1.227	0.960
BOTULIN	4	6	0.722	0.953
AKAP13	7	21	0.787	0.947
SARS	10	45	0.819	0.939
RAB	10	45	0.441	0.937

a The number of gene pairs is equal to *G_m_*(*G_m_*–1)/2, where *G_m_* is the number of genes in the network.

Identifying networks that are tightly regulated in some phenotypes and loosely regulated in others suggests that the level of control across samples in a population may change dramatically, reflecting the nature of the disease process. While identifying changes in tightness of regulation of networks can provide insight into molecular differences between phenotypes, some networks may be tightly regulated in all phenotypes examined. For example, we found that the G-protein signaling (GS) network is the most tightly regulated network in normal prostate (NP), as well as in primary (PT) and metastatic prostate tumors (MT). The GS network comprises major signaling proteins downstream of G-protein coupled receptors, including both the catalytic (*PRKACA*) and regulatory (*PRKAR1A*) subunits of the cAMP-dependent protein kinase C (*PKC*). PKC family members phosphorylate a wide variety of protein targets and are known to be involved in diverse cellular signaling networks, such as those associated with cell adhesion, cell transformation, cell cycle checkpoint, and cell volume control. In 18 NP samples, the pairwise orderings among the six GS network genes matched the corresponding normal prostate rank template identically for all 15 pairs in the network (*μ_R_* = 1.000). Similarly, network rankings in PT samples and MT samples matched the respective templates for 98.9% (*μ_R_* = 0.989) and 99.5% (*μ_R_* = 0.995) of all pairwise orderings on average. We also found that a single network ranking was shared by the majority of NP samples (100%), PT samples (83%), and MT samples (92%); in particular, therefore, the GS network rank template was identical in all three phenotypes. Furthermore, the remaining samples in PT and in MT displayed only a single mismatch in pairwise orderings compared to the template.

There are several possible explanations for observing tight regulation of certain network rankings in a phenotype. In the simplest case, the genes in a network may be expressed at greatly disparate magnitudes, making a change in their relative expression rankings less likely. We can see that this is most likely true for the GS and FOSB networks, both of which displayed the highest rank conservation for all three prostate phenotypes. The average gene-to-gene expression variance across all samples for these networks fell between 1.14–1.58, roughly three times the average gene-to-gene variance for all 248 networks (∼0.41). As such, a change in the relative ordering among genes in these networks would require a more dramatic change in the expression of individual genes. Networks like GS and FOSB are therefore analogous to “housekeeping” genes, as the ranking of genes in each is expected to remain the same in most samples.

**Figure 3:**
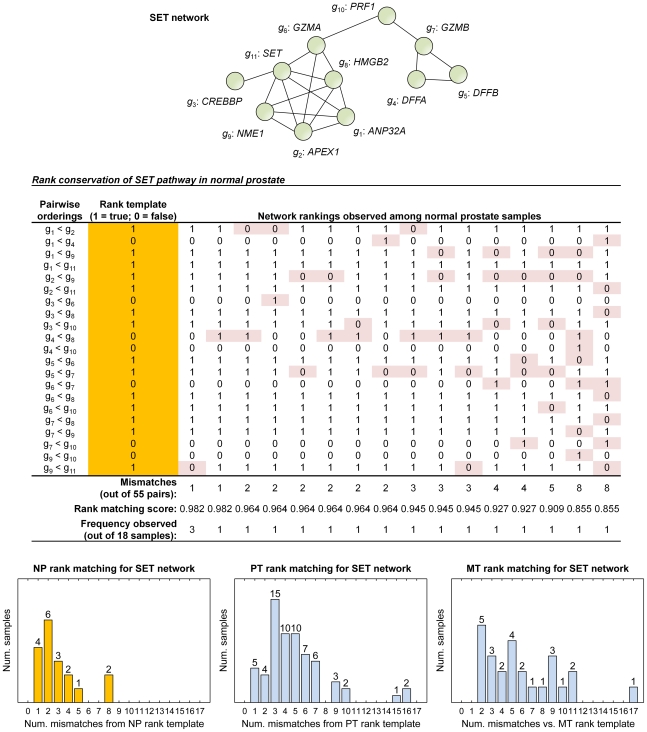
Example of a tightly regulated network in normal prostate. A simplified diagram of the SET network, comprising 11 signaling proteins involved in granzyme mediated apoptosis, is shown in the center. The NP rank template for the network is highlighted yellow, and each unique ranking observed in NP samples is shown to the right with mismatches highlighted red. The histograms at the bottom demonstrate the increased variation in ranking in PT and MT, indicated by greater number of mismatches from the respective rank templates.

Alternatively, small variation in ordering—nearly the same ranking in all samples of the same phenotype—could indicate that a network is critical to maintaining some specific cellular function. This is more likely in cases with less gene-to-gene expression variance within a network; if pairwise orderings can be easily altered by small changes in expression but remain consistent, some force such as selective pressure might drive the cell to minimize fluctuation in the expression of network genes. We found that the SET network is tightly regulated in NP samples, but displays much smaller gene-to-gene variance than networks like GS and FOSB. The SET network—also known as the granzyme mediated apoptosis pathway—comprises a total of 11 genes (illustrated in **Figure 3**), and is an important cytotoxic T cells mechanism for fighting tumors and virus-infected cells [25]. While the SET network displays greater variation in ranking among NP samples than GS or FOSB (*μ_R_* = 0. 945), 16 out of 18 samples show only five or fewer mismatches compared to the 55 pairs in the rank template. We hypothesize that expression of genes within the SET network is highly consistent in NP samples to maintain proper function of cellular defense mechanisms.

Tightly regulated networks in disease phenotypes might also lead to useful hypotheses about cell behavior. The RAN network, similar to SET, is tightly regulated across MT samples, and shows relatively low gene-to-gene variation within the network. The RAN network contains five genes: regulator of chromosome condensation (*CHC1*), Ras-related nuclear protein (*RAN*), RAN binding proteins 1 and 2 (*RANBP1* and *RANBP2*), and RAN GTPase activating protein 1 (*RANGAP1*). In MT samples, on average, the pairwise orderings among the five RAN network genes matched the corresponding MT rank template for 96.0% of all pairs in the network (*μ_R_* = 0.960). This network is involved in the export of mRNA transcripts from the nucleus to the cytosol for subsequent translation. Although it is unclear what advantage tight regulation of the RAN network may confer upon metastatic prostate tumors, there is clearly little variation in network ranking. Importantly, the mutation rates in cancer cells are increased 200-400 fold—providing ample opportunity for changes to be fixed by natural selection or random fixation (if the change is not selectively advantageous or disadvantageous).

We can learn more by examining the tightness of regulation for the same network in different phenotypes. The SET network in PT samples has a rank conservation index equal to 0.909, which is significantly lower than in NP samples (*P*-value<0.05); similarly, *μ_R_* for SET in MT samples is equal to 0.891. As seen in **Figure 3**, the decreased network rank conservation in PT and MT is due to a greater number of samples with rankings different from the respective templates (i.e., more samples with greater numbers of mismatches). The increased variation in network ranking seen in the two stages of prostate cancer might indicate that the biological function associated with SET genes (i.e., granzyme mediated apoptosis) plays a lesser role in behavior of these cells, and is therefore under looser control. It is also possible that in primary and metastatic prostate tumors—obviously more malignant phenotypes compared to normal prostate—the SET network becomes *deregulated* and that this deregulation contributes to its malignancy. Alternatively, an increase in mutation rates with malignancy might have resulted in more random fixations.

These rank conservation indices estimate population statistics based on limited sample sizes (on the order of 20–100, as seen in **Table 2**), and hence some variation is expected even if the true indices were the same. For instance, the difference in the rank conservation index for the SET network between NP and PT or between NP and MT could be a small-sample effect and would need to be validated with either a larger study or by a suitable permutation test (see **Section II** below). However, even these small-sample estimates generate specific hypotheses, such as an increase in disorder in the more malignant phenotype, which can then be meaningfully explored by examining a variety of datasets and phenotypes—discussed in the following sections.

**Table 2:** Human disease gene expression datasets studied with DIRAC.

Dataset	Ref	Samples	Tissue type	Disease/source (subtypes)^a^	Short name^b^	Subtype samples
A	[19]	68	gastrointestinal sarcoma	gastrointestinal stromal tumor	GIST	37
				Leiomyosarcoma	LMS	31
B	[26]	43	ovarian tumors	carcinoma-like ovarian tumor	CL ovarian tumor	20
				adenoma-like ovarian tumor	AL ovarian tumor	23
C	[27]	101	skin fibroblasts	Marfan syndrome subjects	MFS fibroblast	60
				control subjects	non-MFS fibroblast	41
D	[28]	44	head and neck skin cells	head and neck squamous cell carcinoma	HNSCC	22
				normal head and neck skin cells	normal head/neck	22
E	[29]	60	primary breast cancer tumor	patients non-response (cancer recurred) to treatment	(nr) breast cancer	28
				patients responsive (disease-free) to treatment	(r) breast cancer	32
F	[30]	61	dorsolateral prefontal cortex and orbitofrontal cortex	Bipolar disorder patients	bipolar cortex	30
				control patients	non-bipolar cortex	31
G	[31]	72	blood and bone marrow	acute myeloid leukemia	AML 1	25
				acute lymphocytic leukemia	ALL 1	47
H	[32]	48	blood and bone marrow	acute myeloid leukemia	AML 2	24
				acute lymphocytic leukemia	ALL 2	24
I	[24]	83	normal and tumorgenic prostate	primary prostate tumors	(p) prostate cancer	65
				normal prostate tissue	normal prostate	18
J	[24]	43	normal and metastatic prostate	metastatic prostate tumors	(m) prostate cancer	25
				normal prostate tissue	normal prostate	18
K	[24]	90	prostate tumor	metastatic prostate tumors	(m) prostate cancer	25
				primary prostate tumors	(p) prostate cancer	65

a For each set of expression profiles, the two subtypes are listed in order from most to least malignant (e.g., tumor type with worst prognosis or cancer versus control).

b Short names are used to reference specific phenotypes in subsequent figures.

#### Deregulation of network ranking in disease

As described for the SET network above, certain networks may be tightly regulated in one phenotype, but not in another. The SET network appears to be relatively tightly regulated in normal prostate, but more loosely regulated in both primary and metastatic prostate tumors. Cases such as this represent the deregulation of a network in one phenotype relative to another. We used the difference in rank conservation index values between phenotypes as the basis for identifying the most deregulated networks. For example, in comparing NP samples to MT samples, we first calculated the rank conservation index for all networks in both phenotypes. Next, we identified the networks with the greatest absolute difference in index values between NP and PT (i.e., highly conserved in one class but not in the other). Based on sample permutation tests, we found that 67 out of 248 networks had a significant difference in index values (*P*-value<0.05; see **Materials and Methods**). The network with the largest conservation difference—the FIBRINOLYSIS network—was more tightly regulated in NP (*μ_R_* = 0.891) than in MT (*μ_R_* = 0.736) (**Table 3**). The FIBRINOLYSIS network comprises 12 genes and breaks down fibrin clots formed during coagulation. It has previously been reported that patients with metastatic prostate cancer occasionally exhibit enhanced fibrinolytic activities with symptoms of bleeding, epistaxis, or other forms of hemorrhage [33]. Deregulation of the FIBRINOLYSIS in MT samples might therefore be directly linked to malignant features of the disease. However, without further information it is impossible to discern whether loose regulation of this network is a causative mechanism in MT, or occurs as a downstream effect of some other perturbation in tumor progression.

**Table 3:** Most differentially regulated networks between three stages of prostate disease.

Differentially regulated networks (PT vs. NP)						
Network name	Num. genes	Num. gene pairs^a^	*μ_R_* in PT	*μ_R_* in NP	Abs. difference in *μ_R_*	*P*-value
TCRA	12	66	0.859	0.928	0.069	5.85E-04
TCRMOLECULE	5	10	0.871	0.939	0.068	6.69E-04
EIF2	7	21	0.854	0.915	0.061	1.33E-03
TERC	6	15	0.877	0.933	0.056	2.29E-03
NEUTROPHIL	8	28	0.848	0.901	0.053	3.33E-03
GLYCOLYSIS	8	28	0.879	0.929	0.050	4.57E-03
ACE2	11	55	0.835	0.885	0.050	4.72E-03
FIBRINOLYSIS	12	66	0.847	0.891	0.044	9.17E-03
INTRINSIC	22	231	0.852	0.896	0.044	9.45E-03
CLASSIC	10	45	0.886	0.930	0.044	9.74E-03

Differentially regulated networks (MT vs. NP)						
Network name	Num. genes	Num. gene pairs	*ì_R_* in MT	*ì_R_* in NP	Abs. difference in *ì_R_*	*P*-value
FIBRINOLYSIS	12	66	0.736	0.891	0.156	−6.66E-16
EXTRINSIC	12	66	0.716	0.870	0.155	−6.66E-16
INTRINSIC	22	231	0.761	0.896	0.135	2.02E-05
CLASSIC	10	45	0.829	0.930	0.100	2.90E-04
TERC	6	15	0.843	0.933	0.091	6.21E-04
ION	5	10	0.892	0.806	0.086	8.35E-04
COMP	14	91	0.832	0.914	0.082	1.20E-03
NEUTROPHIL	8	28	0.819	0.901	0.082	1.21E-03
ARF	15	105	0.829	0.911	0.081	1.32E-03
PEPI	5	10	0.808	0.889	0.081	1.34E-03

Differentially regulated networks (MT vs. PT)						
Network name	Num. genes	Num. gene pairs	*μ_R_* in MT	*μ_R_* in PT	Abs. difference in *μ_R_*	*P*-value
EXTRINSIC	12	66	0.716	0.856	0.140	−6.66E-16
FIBRINOLYSIS	12	66	0.736	0.847	0.111	8.06E-06
INTRINSIC	22	231	0.761	0.852	0.091	4.03E-05
ION	5	10	0.892	0.803	0.089	6.05E-05
PEPI	5	10	0.808	0.895	0.087	6.85E-05
ARGININEC	6	15	0.880	0.960	0.080	1.65E-04
LEPTIN	8	28	0.807	0.727	0.080	1.73E-04
NOTCH	4	6	0.853	0.931	0.077	2.42E-04
PLC	8	28	0.800	0.859	0.059	1.74E-03
BETAOXIDATION	6	15	0.864	0.922	0.058	1.86E-03

a The number of gene pairs is equal to *G_m_*(*G_m_*–1)/2, where *G_m_* is the number of genes in the network.

**Figure 4:**
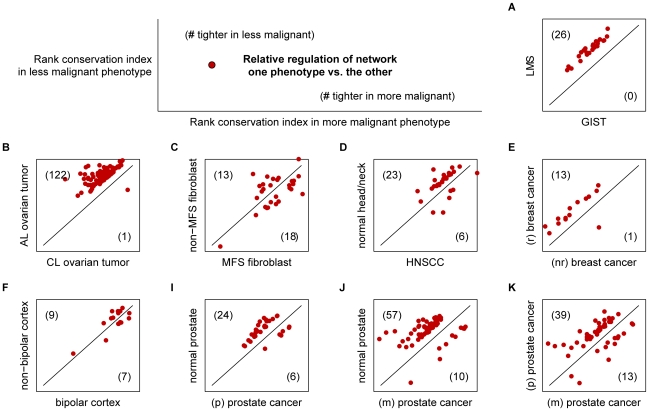
Deregulation of networks in disease. For each dataset, networks were selected according to the greatest absolute difference in rank conservation between the two phenotypes. Using this subset of networks, the rank conservation index values in the less malignant phenotype (y-axis) were plotted against indices in the more malignant phenotype (x-axis). Higher rank conservation in the less or more malignant phenotypes is indicated by points above or below the diagonal line, respectively. Panel labels (**A**–**K**) correspond to datasets listed in **Table 2**.

Upon inspecting the remaining differentially regulated networks between NP and MT, we found that in fact, 57 out of 67 significantly deregulated networks identified showed tighter regulation in NP than in MT (**Figure 4J**). The strong majority of networks more tightly regulated in the NP (*P*-value = 5.14×10^−8^ from a binomial distribution; see **Table 4**) lends evidence to the theory that deregulation of network ranking is in some way related to increased malignancy. As such, the DIRAC approach may be useful both in the stratification of disease and/or in assessment of the progression of disease. To explore this hypothesis further, we examined a number of gene expression datasets available for public download from the NCBI Gene Expression Omnibus (**Table 2**). These datasets included expression profiles from multiple cancers such as breast, ovarian, and blood (leukemia), as well as diseases of the brain/nervous system, skin, and intestinal tract (note: the leukemia datasets G and H were excluded from this particular comparison, as we found no clear evidence for which subtype—AML or ALL—is more malignant). We repeated the procedure described for NP and MT for each binary phenotype comparison from the expression data. In all but one case out of nine, the less malignant phenotype had a greater number of high conserved (tightly regulated) networks (**Figure 4**). That is, a network appears much more likely to become deregulated in worse cases of disease. Importantly, the dataset for the one exception—comparing Marfan syndrome and normal fibroblasts—contained expression values for only ∼4,000 genes (compared to 20,000 or more in most of the other datasets). Due to the small number of genes, many of the networks contained significant gaps, which may have produced less robust results. Still, the overall trend seen in **Figure 4** suggests that in malignant phenotypes, networks are often more loosely regulated, with greater variation in expression ranking of participating genes from sample to sample. The global pattern of increased disorder with malignancy highlights the utility of studying gene expression ordering within networks, and also reveals a striking phenomenon that could drive future investigation and may lead to new understandings of gene expression in disease.

**Table 4:** Statistical significance of network deregulation in malignant phenotypes.

Dataset	# tighter in less malignant	# tighter in more malignant	Outcome	Binomial *P*-value
A	26	0	1	0.00
B	122	1	1	0.00
C	13	18	0	0.76
D	23	6	1	0.00
E	13	1	1	6.10E-05
F	9	7	1	0.23
I	24	6	1	1.62E-04
J	57	10	1	3.41E-10
K	39	13	1	6.38E-05
**Total**	326	62	8	0.00
		**Binomial ** ***P*** **-value for outcomes:**	0.002	

#### Global regulation of networks across phenotypes

Averaging rank conservation indices over all the networks provides a measure of global regulation of networks in different phenotypes. For example, networks in normal prostate are more highly conserved on average (0.903) than networks in metastatic prostate cancer (0.884). This difference suggests that the more malignant cancer subtype (MT) may have greater overall variation in network rankings among different samples. We used the gene expression datasets described above to compare global regulation of network rankings among a number of phenotypes. For each phenotype, we calculated rank conservation indices for all networks and used the average conservation as a rough measure of how tightly or loosely regulated networks *tend* to be in each case.

**Figure 5:**
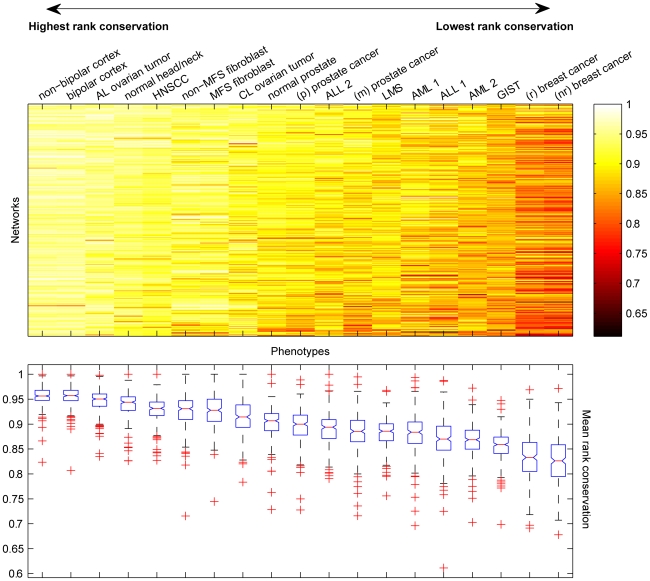
Diverse rank conservation of networks across phenotypes. Colors on the heatmap represent rank conservation indices for each network in 19 different phenotypes, where brightest indicates very tight regulation of network ranking in a phenotype and darkest indicates loose regulation of networks, with greater shuffling of gene rankings.

We used the average index value to order phenotypes from highest to lowest global conservation. Phenotypes with the highest average conservation primarily have tightly regulated networks across samples in the population. For example, most networks in non-bipolar cortex and bipolar cortex were found to have conservation index values greater than 0.95 (seen as bright colors on the heatmap in **Figure 5**) for average values of 0.9561 and 0.9556, respectively. In contrast, many networks in the two breast cancer phenotypes (r—responsive to treatment; nr—non-responsive to treatment) have rank conservation indexes less than 0.80 (dark colors on the **Figure 5** heatmap). In this case, the low global conservation—average index values of 0.835 and 0.826 in (r) breast cancer and (nr) breast cancer, respectively—suggests that network rankings in these disease phenotypes have looser regulation and greater variation. Based on a one-way ANOVA, the estimated overall *P*-value for the ordering of phenotypes in **Figure 5** is zero.

Interestingly, the trend of lower conservation in more malignant phenotypes described in the previous section seems to persist even from a coarser, global perspective. For example, networks in the less malignant adenoma-like ovarian tumors are more highly conserved on average (0.947) than in more malignant carcinoma-like ovarian tumors (0.913). The same was seen when examining all three prostate phenotypes, where normal prostate is more tightly regulated overall than primary (p) prostate cancer, which itself is more tightly regulated than metastatic (m) prostate cancer. Even for the most highly conserved phenotypes (non-bipolar and bipolar cortex) and lowest conserved phenotypes (breast cancers), networks are more tightly regulated on average in the less malignant phenotype of each pair. We also observed interesting differences observed based on tissue-type, where expression ranking of networks in brain and ovarian tissue displayed higher conservation on average than prostate tissue, which is in turn is more highly conserved than in blood and in breast tissue. Thus, at least two global trends must be considered in evaluating network deregulation: (i) the severity of the disease, and (ii) the tissue of origin.

### Sample-Level DIRAC

In order to identify variably expressed networks between two selected phenotypes, we designed a rank difference score (Δ), calculated for each sample based on rank matching scores. For a particular network, this measure indicates the similarity between the ordering of network genes in a sample to the template of one class versus the template of the other. The difference score ranges from -1 to 1, with positive values suggesting the first phenotype, and negative values suggesting the second, culminating in simple rules for classifying an expression profile. Our purpose in introducing the rank difference score was two-fold: (i) to identify variably expressed networks between two selected phenotypes; and (ii) to validate the DIRAC approach to network identification, and the emphasis on combinatorial interactions, by demonstrating the discriminative power of the networks identified.

#### Variably expressed networks in normal prosate and cancerous prostate

**Figure 6:**
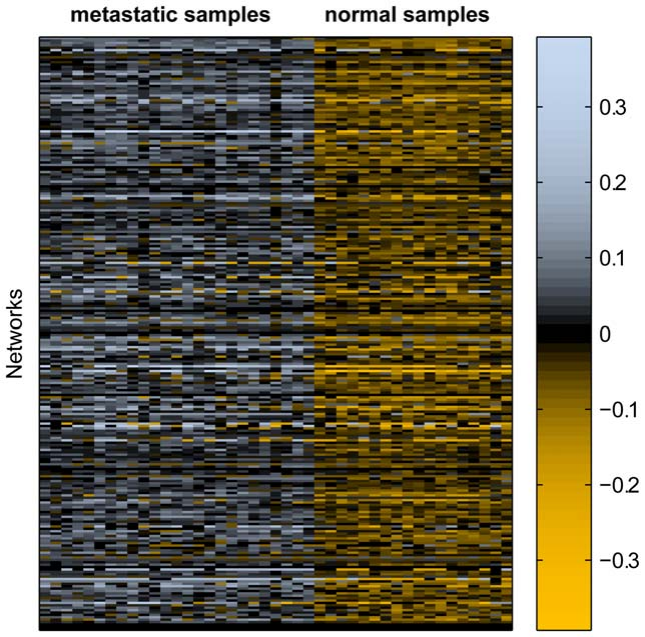
Differential rank conservation across all networks for a set of two prostate phenotypes. Positive rank difference scores predict a metastatic sample and negative difference scores predict a sample as normal.

As shown in **Figure 6**, the positive versus negative trend holds for most samples in MT and NP across all networks. To determine the most variably expressed networks between MT and NP, we (i) defined rank templates for each phenotype; (ii) calculated rank matching scores and evaluated the rank difference score for each sample; and (iii) chose the networks with the largest estimated classification rate. Specifically, the classification rate for a network is defined as the average of sensitivity and specificity for predicting sample classes in the training data (i.e., apparent accuracy).

To evaluate whether variably expressed networks represented meaningful differences between MT and NP gene expression profiles, we used permutation-based testing to assess the statistical significance of estimated network classification rates (see **Materials and Methods**). A total of 176 networks significantly discriminated between expression profiles of MT and NP (*P*-value<0.05), the top ten of which appear in **Table 5**. Among these differentially expressed networks, we estimated that only 6.7% (between 11 and 12 of the 176 total) are likely to have been found by chance rather than based on true differences between the phenotypes, as determined by the FDR.

**Table 5:** Most variably expressed networks between different stages of prostate cancer.

Variably expressed networks (PT vs. NP)					
Network name	Num. genes	Num. gene pairs^a^	Template difference^b^	Apparent accuracy	*P*-value
KERATINOCYTE	46	1035	0.070	0.981	<1.0E-07
TOLL	31	465	0.073	0.945	1.21E-05
MAPK	83	3403	0.064	0.941	2.02E-05
MET	35	595	0.103	0.941	2.02E-05
FCER1	36	630	0.059	0.931	6.85E-05
INTEGRIN	34	561	0.094	0.923	1.21E-04
AT1R	34	561	0.096	0.922	1.25E-04
ERK	29	406	0.037	0.921	1.29E-04
CARDIACEGF	17	136	0.118	0.920	1.33E-04
IL1R	28	378	0.071	0.915	1.81E-04

Variably expressed networks (MT vs. NP)					
Network name	Num. genes	Num. gene pairs	Template difference	Apparent accuracy	*P*-value
MAPK	83	3403	0.111	1.000	<1.0E-07
DEATH	29	406	0.128	1.000	<1.0E-07
IL2RB	35	595	0.096	1.000	<1.0E-07
HIVNEF	53	1378	0.148	1.000	<1.0E-07
MET	35	595	0.165	1.000	<1.0E-07
NO1	27	351	0.125	1.000	<1.0E-07
NFAT	47	1081	0.164	1.000	<1.0E-07
PPARA	50	1225	0.100	1.000	<1.0E-07
ACTINY	19	171	0.123	1.000	<1.0E-07
FCER1	36	630	0.111	0.990	<1.0E-07

Variably expressed networks (MT vs. PT)					
Network name	Num. genes	Num. gene pairs	Template difference	Apparent accuracy	*P*-value
FCER1	36	630	0.119	0.985	<1.0E-07
TCR	44	946	0.103	0.969	<1.0E-07
BCR	33	528	0.133	0.969	<1.0E-07
HIVNEF	53	1378	0.119	0.969	<1.0E-07
MET	35	595	0.126	0.969	<1.0E-07
PDGF	27	351	0.128	0.957	<1.0E-07
BIOPEPTIDES	37	666	0.107	0.957	<1.0E-07
MAPK	83	3403	0.100	0.954	<1.0E-07
IL2RB	35	595	0.087	0.954	<1.0E-07
AT1R	34	561	0.111	0.954	<1.0E-07

a The number of gene pairs is equal to *G_m_*(*G_m_*–1)/2, where *G_m_* is the number of genes in the network.

b The template difference represents the Hamming distance between two binary rank template vectors.

**Figure 7:**
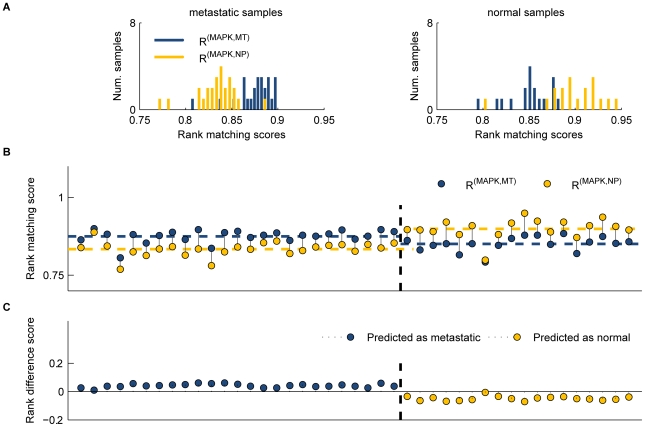
Differential rank conservation of the MAPK network in metastatic prostate cancer and normal prostate. (**A**) Histograms of rank matching scores. MT template matching scores (*R* ^(MAPK,MT)^) are higher on average in MT samples than NP matching scores (*R* ^(MAPK,NP)^). In NP samples, *R* ^(MAPK,NP)^ scores are higher on average than *R* ^(MAPK,MT)^ scores. (**B**) Rank matching scores for the MAPK network. Comparing the two rank matching scores in each sample, MT samples are more similar to the MT template than to the NP template in all cases; NP samples are ranked more similarly to the NP template more than the MT template in all cases. (**C**) Rank difference score values for the MAPK networks. Samples are classified as MT if the rank difference score is greater than zero and as NP if the difference is less than zero.

The principal features governing the rank difference score, and also an example of its application to molecular classification, are illustrated in **Figure 7** for the MAPK network, which we identified as one of the most differentially expressed networks between normal prostate and metastatic prostate tumors. Here, *R*(**x** *_n_*) denotes the rank matching score for a profile **x** *_n_*, and superscripts indicate the network and phenotype of the rank template (e.g., *R* ^(MAPK,MT)^(**x** *_n_*) represents the rank matching score for a sample when compared to the ordering defined in the MT template). The rank difference score is the difference in matching score values for a particular sample: *R* ^(MAPK,MT)^(**x** *_n_*)–*R* ^(MAPK,NP)^(**x** *_n_*). This measure captures low variance of network ranking within phenotypes, but disparate rankings between phenotypes. The rank difference values calculated for the MAPK network for all samples are shown in **Figure 7**, along with the corresponding phenotype predictions (i.e., MT where positive, NP if negative). Interestingly, MAPK signaling has been previously reported to be involved in the cancerous transformation of prostate cells [34], [35].

#### DIRAC-based classification of disease phenotypes

**Figure 8:**
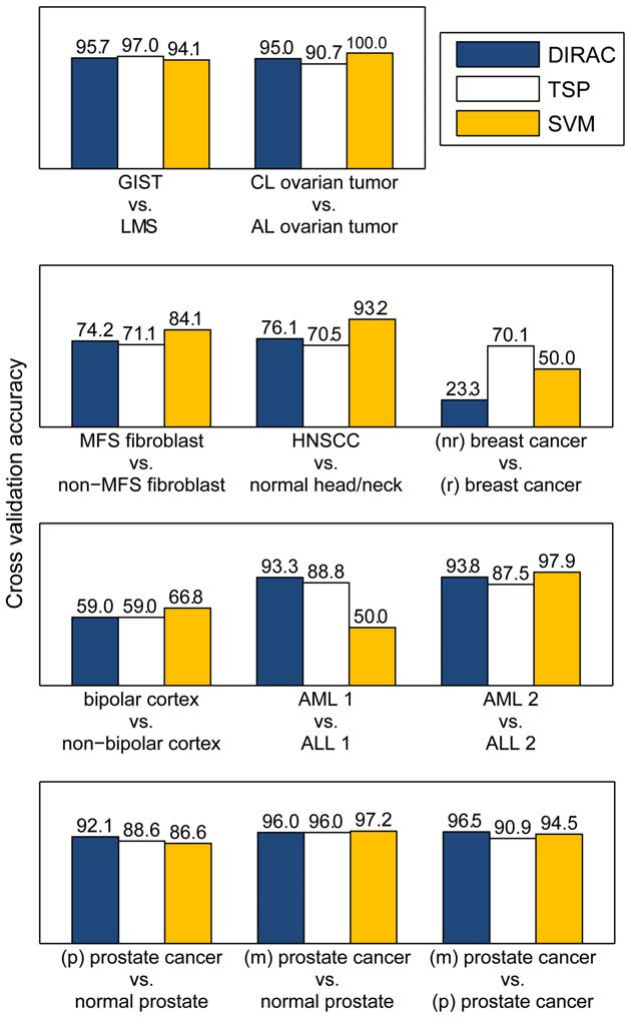
Classification with DIRAC compared to other methods.

The top networks selected by DIRAC based on the difference score (i.e., the single best network for separating each different pair of phenotypes) were used to classify gene expression profiles in cross-validation. Specifically, we used leave-one-out cross-validation (LOOCV) to estimate how accurately the top networks selected could predict the phenotype of future samples (**Figure 8**). Importantly, all processes including defining rank templates, calculating rank difference scores, and selecting the best network were done within cross-validation, using only the training samples (i.e., no information from test samples was used to train classifiers). For comparison, we selected the top *G_m_* differentially expressed genes—where *G_m_* is equal to the number of genes in the top network selected by DIRAC—and used the top-scoring pair (TSP) algorithm [22], [36] and support vector machines (SVM) [37], [38] to classify samples in each of the datasets. We found that our method performed well in a number of the datasets, with estimated accuracies between 92–96% in gastrointestinal sarcoma, ovarian cancer, leukemia, and prostate cancer—including comparisons between normal prostate and cancer as well as different stages of prostate cancer (**Figure 8**). In cases with poor accuracies, such as responsiveness of breast cancer to therapy, bipolar disorder, and Marfan syndrome, we observed that other methods also failed to accurately classify samples, suggesting that these phenotypes are inherently difficult to separate based on the available expression data.

Overall, we found that classification, when restricted to only the genes in the top network (as determined by DIRAC), is nearly as accurate as using the overall *G_m_* most differentially expressed genes (in TSP or SVM). Our foremost goal was not to propose a new classifier, but to aid in biological discovery and hypothesis generation; the classification accuracy simply affirms the robustness of the network rank regulation measure. Specifically, the classification experiment validates DIRAC by demonstrating the importance of combinatorial interactions: the potential loss of discriminating power in individual genes is countered by discriminating interactions.

### Implications for Systems Medicine

Systems medicine approaches assume that disease arises from disease-perturbed biological networks in the relevant organ or organs. These disease-perturbed networks alter the envelopes of information that they express—and these changes encode the pathophysiology of the disease. Moreover, the altered patterns of information can elucidate new strategies for diagnosis or therapy. Future drugs will likely be designed to re-engineer disease-perturbed networks to behave in a more normal fashion, or at least to abrogate their most deleterious consequences. This will require a new drug target identification approach, and re-engineering disease-perturbed networks appropriately will almost always require multiple drugs. Likewise, the perturbed nodal points in disease-perturbed networks can be expressed as proteins in the blood—where the disease-altered levels of expression may reflect the disease process. These disease-altered blood proteins will create unique blood fingerprints specific for each disease process, and thus provide powerful diagnostics. These advances rely upon the proper identification of disease-perturbed networks. To date, most of the evaluation of networks has employed lists of transcripts that are perturbed from the levels of their counterparts in normal organs. This listing, as with genome-wide association (GWAS) studies, misses the key fact that disease-perturbed networks must be assessed in the context of the combinatorial interactions of their nodal components.

Our method is the first approach that begins to account for the combinatorial behavior of interacting genes, mRNAs and/or proteins. Using DIRAC-based calculations allows us to begin to assess the key disease-perturbed networks that may aid in the approach to diagnosis and therapy. We also stress that these methods will almost certainly prove powerful in the stratification of disease types. The example of gastrointestinal stromal tumors (GIST) and leiomyosarcomas (LMS), histologically indistinguishable, but clearly classifiable by a primitive version of DIRAC, is striking. We believe this will be a powerful approach in, for example, distinguishing various types of neurodegenerative diseases, as well as the stratification of complex diseases such as Alzheimer's. Notably exciting, some of the key transcripts used in this classification process actually encoded proteins secreted into the blood. Findings of this nature could lead to the use of altered blood levels of proteins for diagnosis without the need to sample disease tissues. Emerging technologies will make these measurements possible at the single cell level, exposing other exciting possibilities for diagnosis using the strategies outline above. We predict the application of DIRAC as a powerful clinical tool in the advancing proactive, rather than reactive, new medicine—the so-called P4 medicine (predictive, personalized, preventive and participatory)—where blood and single-cell diagnostics will be the foundation of the P4-medicine revolution.

### Conclusions

In this study we demonstrate a novel method to identify highly discriminative biological networks based on differing patterns of gene expression ranking within networks. These results provide a coarse, but meaningful, glimpse into patterns of network regulation for different phenotypes based on combinatorial relationships between the involved genes. For example, when comparing two disease states, it appears to be very common (although not universal) for network rankings to be more varied—or less tightly regulated—in the more pathological state. This increased disorder associated with malignancy might be expected, as mutations and other altered behavior of biomolecules lead to breakdown of typical functioning in biological networks; rank conservation index values calculated in DIRAC represent a quantitative means to study and further verify this notion. Importantly, this method not only identifies perturbed networks, but does so in such a way that it can classify samples. Thus, predictive accuracy becomes a strong measure for the validity of the perturbed network as a reproducible hallmark of the disease phenotype. Such high predictive accuracy in classification adds much stronger evidence that biologically meaningful network differences are found than only a low *P*-value or FDR, which simply measure how likely the result derives from chance. Measures of global regulation can also give useful information for designing research to identify expression-based classifiers of disease. For instance, it would be more fruitful to search for clear molecular signatures with tightly regulated phenotypes. In cases with mostly loosely regulated networks, the greater variation from sample-to-sample would pose a more difficult challenge for identifying reliable classifiers. Studying rank regulation of biologically relevant networks thus offers a promising tool for measuring network behavior within and across different populations. Looking forward, the results obtained through this approach should provide increased insight into phenotypic processes of importance in biology and medicine.

## Materials and Methods

The methods and analyses presented here were performed entirely in Matlab. Source code files are available for download at http://www.igb.uiuc.edu/labs/price/downloads.

### Microarray Data

Given the list {*g*_1_, …, *g_Gm_*} of *G_m_* genes within a network *m* on a microarray, we let **X** = (*X*_1_, …, *X_Gm_*) denote the corresponding expression profile, where *X_i_* is the expression level of gene *g_i_*. Our data then consists of a *G_m_* x *N* matrix; the *n* ^th^ column represents the expression profile **x** *_n_* of the *n* ^th^ sample, *n* = 1, …, *N*. In addition, each sample is labeled by a phenotype *Y*∈{A, B, …, *K*}. The labeled training set is *F* = {(**x**_1_, y_1_), …, (**x** *_N_*, y*_N_*)}. Expression profiles **X** and phenotype labels *Y* are regarded as random variables, and the elements of *F* represent independent and identically distributed samples from some underlying probability distribution of (**X**, *Y*).

Our analysis is based entirely on the *ranks* within each expression profile. With *G_m_* genes, there are *G_m_*! possible orderings for the expression values. The networks we consider typically have tens or hundreds of genes; consequently, working directly with individual permutations is not feasible. For example, any estimated distribution over permutations using training data would be highly singular. Instead, we base the analysis entirely on pairwise comparisons.

### Rank Template Matching for Networks

Knowing the ordering of the gene expressions within each network expression profile is equivalent to knowing all of the pairwise orderings, i.e., whether *X_i_*<*X_j_* or *X_i_*>*X_j_* for each distinct pair of genes 1≤*i*, *j*≤*G_m_* within the network *m*. Evidently, there are *G_m_*(*G_m_*–1)/2 such pairs. For example, if there are *G_m_* = 4 genes, then there are six distinct ordered pairs: {(1, 2), (1, 3), (1, 4), (2, 3), (2, 4), (3; 4)}. In order to define a template representing the expected ranking of network genes within a phenotype, we consider the probabilities Pr(*X_i_*<*X_j_* |*Y* = *k*) for each pair of genes *g_i_*<*g_j_* and for each phenotype *k*. We estimate these probabilities from the training set by computing the fraction of samples in each phenotype for which gene *g_i_* is expressed less than gene *g_j_*. The rank template for a fixed network *m* and phenotype *k* is the binary vector *T* ^(*m*,*k*)^ of length *G_m_*(*G_m_*–1)/2 where the *i*,*j* ^th^ component is 1 if Pr(*X_i_*<*X_j_* |*Y* = *k*)>0.5 and 0 if Pr(*X_i_*<*X_j_* |*Y* = *k*)≤0.5. The calculation of a rank template is illustrated in **Figure 1**.

Given an expression profile **x** *_n_* for the network *m*, there is then a natural measure for how well the sample matches the template *T* ^(*m*,*k*)^. The rank matching score of sample *n* is denoted by *R* ^(*m*,*k*)^(**x** *_n_*) and is defined to be the fraction of the *G_m_*(*G_m_*–1)/2 pairs for which the observed ordering within **x** *_n_* matches the template—the orderings expected for phenotype *k*. See **Figure 1** for an illustration of a rank matching score.

### Rank Conservation Indices

Averaging the rank matching score over all the samples in a phenotype *k* yields a rank conservation index denoted by *μ_R_* ^(*m*,*k*)^ = *E*(*R* ^(*m,k*)^|*Y* = *k*). This index is estimated by averaging the scores *R* ^(*m*,*k*)^(**x**) over all the samples (**x**, *y*) in the training set for which *y* = *k*. Whereas the rank matching score is a sample-based statistic, i.e., it is defined for each expression profile, the rank conservation index is a population statistic. The rank conservation index can be seen as a measure of the stability in rankings among the network genes in the phenotype. Two extreme cases correspond to (i) pure random shuffling of the expression values in the phenotype from sample to sample, in which case *μ_R_* ^(*m*,*k*)^≈0.5; and (ii) all samples displaying exactly the same ordering, in which case *μ_R_* ^(*m*,*k*)^≈1. In general, there are many gene pairs *g_i_* and *g_j_* which are expressed on different scales, and hence *x_i_*<*x_j_* across nearly all samples and phenotypes. As a result, one generally finds *μ_R_* ^(*m*,*k*)^≫0.5. This index is similar to entropy in the sense that values of *μ_R_* ^(*m*,*k*)^≪1 indicate a highly disorganized state in which there is a great deal of variation among the rankings in phenotype *k* from sample to sample and values of *μ_R_* ^(*m*,*k*)^≈1 indicate a highly ordered state in which samples have very similar, and hence predictable, orderings among the genes.

### Rank Difference Scores

Consider two phenotypes *Y* = A, B, and a fixed network *m*. If network *m* is tightly regulated in one phenotype, the samples from that phenotype, say *Y* = A, will have high *R* ^(*m*,A)^ values on average. But if *μ_R_* ^(*m*,*k*)^ is large for both *k* = A and *k* = B, and if the two rank templates *T* ^(*m*,A)^ and *T* ^(*m*,B)^ are significantly different, then the samples from phenotype *Y* = A will generally have low values for the statistic *R* ^(*m*,B)^ as well as high values for the statistic *R* ^(*m*,A)^, and vice-versa for the samples from phenotype *Y* = B. We capture this phenomenon, namely low variance of network ranking within a phenotype, but high variance between phenotypes, with a single statistic calculated for each sample: the difference Δ^(*m*)^(**x** *_n_*) = *R* ^(*m*,A)^(**x** *_n_*)–*R* ^(*m*,B)^(**x** *_n_*). Clearly, –1≤Δ^(*m*)^(**x** *_n_*)≤1 with positive (respectively, negative) values providing evidence that the phenotype of sample *n* is *Y* = A (resp., *Y* = B). As a result, the difference score provides a classifier for phenotype identification based on the degree of regulation of the genes in network *m*. A new sample *n* is predicted to belong to phenotype *Y* = A if Δ^(*m*)^(**x** *_n_*)>0 and to phenotype *Y* = B if Δ^(*m*)^(**x** *_n_*)≤0. The classification rate for network *m* is then: *η*(*m*) = Pr(Δ^(*m*)^(**X**)>0|*Y* = A)*Pr(*Y* = A)+Pr(Δ^(*m*)^(**X**)≤0|*Y* = B)*Pr(*Y* = B). The calculation of a rank difference score was shown in **Figure 1**.

For example, if *Y* = A denotes prostate cancer and *Y* = B denotes normal prostate, and if we assume that the two phenotypes are *a priori* equally likely, then *η*(*m*) is simply the average of sensitivity and specificity relative to identifying cancer. In order to determine the most variably expressed networks between two given phenotypes, we calculate rank templates for each phenotype, evaluate the differential score for each sample in the training set and choose the networks with the largest estimated classification rate.

One previously reported method, *k*-TSP, classifies expression profiles based on *k* pairs of genes with the most significant expression reversals among all assayed genes [22]. The classifier based on the rank difference score is also based on *k* pairs of genes, with *k* equal to the distance between the two rank templates. To see this, notice that upon computing the difference Δ^(*m*)^(**x** *_n_*) for pathway *m* and phenotypes A and B, the gene pairs (*i*,*j*) for which *T* ^(*m*,A)^(*i*,*j*) = *T* ^(*m*,B)^(*i*,*j*) cancel out. The DIRAC-based classifier therefore reduces to voting among the gene pairs whose probabilities straddle 0.5—i.e., satisfy Pr(*X_i_*<*X_j_* |*Y* = A)<0.5<Pr(*X_i_*<*X_j_* |*Y* = B) or vice versa. However, these *k* pairs of genes are those in the “top-scoring network” as determined by DIRAC rather than the most discriminating *k* pairs overall (as would be identified by *k*-TSP).

### Significance Testing

Procedures for estimating statistical significance are described below for metastatic prostate tumors (MT) and normal prostate (NP). Identical procedures were used for all binary phenotype datasets studied.

#### Deregulated networks based on the difference in rank conservation indices

Under the null hypothesis that no systematic difference in gene expression profiles exists between MT and NP, (i) the original phenotype labels were randomly re-assigned to samples, and rank conservation indices were calculated for all networks in each phenotype; (ii) the absolute difference in rank conservation index values between the two phenotypes was calculated for each network (i.e., *θ*(*m*) = |*μ_R_* ^(*m*,MT)^–*μ_R_* ^(*m*,NP)^| for the *m* ^th^ network); (iii) the first two steps were repeated for 1,000 permutations to generate a null distribution of rank conservation difference values; and (iv) the significance level for *θ*(*m*) representing deregulation of a network between MT and NP was measured as the probability of observing differences in rank conservation greater than or equal to *θ*(*m*) in the null distribution.

#### Classification rate for networks based on the rank difference score

Under the null hypothesis that no systematic difference in gene expression profiles exists between MT and NP, (i) the original phenotype labels were randomly re-assigned to samples, and rank difference scores were calculated for each sample in all networks; (ii) sample classes in the permuted dataset were predicted as MT or NP based on whether the difference score was positive or negative, respectively, and scores were assigned to each network as measured by the estimated classification accuracy (i.e., *η*(*m*) for the *m* ^th^ network); (iii) the first two steps were repeated for 10,000 permutations to generate a null distribution of network classification rates; and (iv) the significance level for the *η*(*m*) in predicting MT and NP profiles was measured as the probability of observing classification rates greater than or equal to *η*(*m*) in the null distribution. To address the issue of multiple-hypothesis testing, we also estimated the false discovery rate (FDR) for each significance level, representing the fraction of our selected features which we would expect to be false positives.

### Evaluating Classification Performance

We used leave-one-out cross validation to estimate the (generalization) error rate of each classification method studied. Importantly, for each classification method tested, all processes were done using only the training samples without including any information from the test sample. Within each iteration of the cross validation loop, expression profiles in the original training data *F* = {(**x**_1_, y_1_), …, (**x** *_N_*, y*_N_*)} are divided into two groups: a training set (*F_train_*) and a test set (*F_test_*). The classifier is trained on the *N*–1 samples of *F_train_* and then used to predict the phenotype of the remaining “left out” sample in *F_test_*. The overall cross validation classification rate after *N* total train-test divisions and predictions is calculated as the average of sensitivity and specificity. Details for training and testing with each type of classifier are described below.

#### DIRAC

Rank templates, rank matching scores, and rank difference scores are calculated uniquely for each new instance of the training set *F_train_*. The single best network is chosen based on the classification rate for samples of *F_train_*, and the rank templates for this network are then used to assign two rank matching scores to the remaining sample comprising *F_test_*. If the difference in matching scores is positive, the sample is predicted to be of phenotype A, otherwise it is classified as phenotype B.

#### TSP

The top-scoring pair (TSP) algorithm is described in detail elsewhere [22]. Here, we first filtered the total number of transcripts in *F_train_*, keeping only the top *G_m_* most differentially expressed genes (DEGs), where *G_m_* is equal to the number of genes in the best network selected by DIRAC. The top features (i.e., DEGs) were selected based on the Wilcoxon ranksum test. Searching among all possible pairwise combinations of genes in the reduced dataset, we identified a single best pair (*X_i_* and *X_j_*) for which the difference |Pr(*X_i_*<*X_j_* | A)–Pr(*X_i_*<*X_j_* | B)| is maximized (or alternatively, |Pr(*X_i_*>*X_j_* | A)–Pr(*X_i_*>*X_j_* | B). The phenotype of *F_test_* is then predicted by comparing the expression levels for this gene pair.

#### SVM

Prior to training a support vector machine (SVM) classifier on the samples of *F_train_*, we also filtered down to the top *G_m_* DEGs within each cross validation loop, where *G_m_* is equal to the number of genes in the best network selected by DIRAC. The SVM was then trained on the expression values of these *G_m_* genes using a Gaussian kernel, and then used to predict the phenotype of *F_test_*.

## Supporting Information

Table S1 Increasing network completeness with NCBI gene name information.
(0.01 MB PDF)
