# Supplementary material for: Identifying Tightly Regulated and Variably Expressed Networks by Differential Rank Conservation (DIRAC)
Source: PLoS Comput Biol. 2010 May 27;6(5):e1000792. doi: 10.1371/journal.pcbi.1000792 (PMC2877722; doi:10.1371/journal.pcbi.1000792)
Supplement: Table S1 — Increasing network completeness with NCBI gene name information. (0.01 MB PDF) [file pcbi.1000792.s001.pdf]

Table S1. Increasing network completeness with NCBI gene name information.

BioCarta network mapping statistics before replacing dataset gene names with NCBI synonyms:

					**Num. networks equal to or above x% completion**					
**Dataset**	**Num. dataset genes**	**Num. network genes**	**Num. matched**	**% matched network genes**	**100%**	**90%**	**80%**	**70%**	**60%**	**50%**
A	30,761	1,296	1,241	0.96	155	228	243	246	247	248
B	9,724	1,296	1,052	0.81	60	127	204	228	239	246
C	3,983	1,296	622	0.48	1	1	6	28	90	169
E	20,021	1,296	1,105	0.85	37	101	208	233	244	247
D	9,724	1,296	1,052	0.81	60	127	204	228	239	246
F	14,941	1,296	1,140	0.88	74	173	225	238	245	248
G	5,600	1,296	908	0.70	25	61	150	195	227	244
H	9,101	1,296	1,077	0.83	70	143	217	233	240	247
I,J,K	9,724	1,296	1,052	0.81	60	127	204	228	239	246

BioCarta network mapping statistics after replacing dataset gene names with NCBI synonyms:

					**Num. networks equal to or above x% completion**					
**Dataset**	**Num. dataset genes**	**Num. network genes**	**Num. matched**	**% matched network genes**	**100%**	**90%**	**80%**	**70%**	**60%**	**50%**
A	30,761	1,296	1,279	0.99	201	245	248	248	248	248
B	9,724	1,296	1,130	0.87	95	178	230	239	247	248
C	3,983	1,296	681	0.53	2	2	17	51	125	195
D	9,724	1,296	1,130	0.87	95	178	230	239	247	248
E	20,021	1,296	1,193	0.92	66	161	235	247	248	248
F	14,941	1,296	1,224	0.94	129	222	244	247	248	248
G	5,600	1,296	974	0.75	42	97	182	217	237	245
H	9,101	1,296	1,134	0.88	97	181	233	241	245	248
I,J,K	9,724	1,296	1,130	0.87	95	178	230	239	247	248
